# Metal-Complexes Bearing Releasable CO Differently Modulate Amyloid Aggregation

**DOI:** 10.1021/acs.inorgchem.3c01522

**Published:** 2023-06-20

**Authors:** Sara La Manna, Valentina Roviello, Fabiana Napolitano, Anna Maria Malfitano, Vittoria Monaco, Antonello Merlino, Maria Monti, Konrad Kowalski, Łukasz Szczupak, Daniela Marasco

**Affiliations:** † Department of Pharmacy, University of Naples “Federico II”, 80131 Naples, Italy; ‡ Department of Chemical, Materials, and Industrial Production Engineering (DICMaPI), University of Naples Federico II, 80125 Naples, Italy; § Department of Translational Medical Science, University of Naples “Federico II”, 80131 Naples, Italy; ∥ Department of Chemical Sciences, University of Naples “Federico II”, 80126 Naples, Italy; ⊥ CEINGE Biotecnologie Avanzate S.c.a r.l. “Franco Salvatore”, 80131 Naples, Italy; # Faculty of Chemistry, Department of Organic Chemistry, University of Łódź, Tamka 12, 91-403 Łódź, Poland

## Abstract

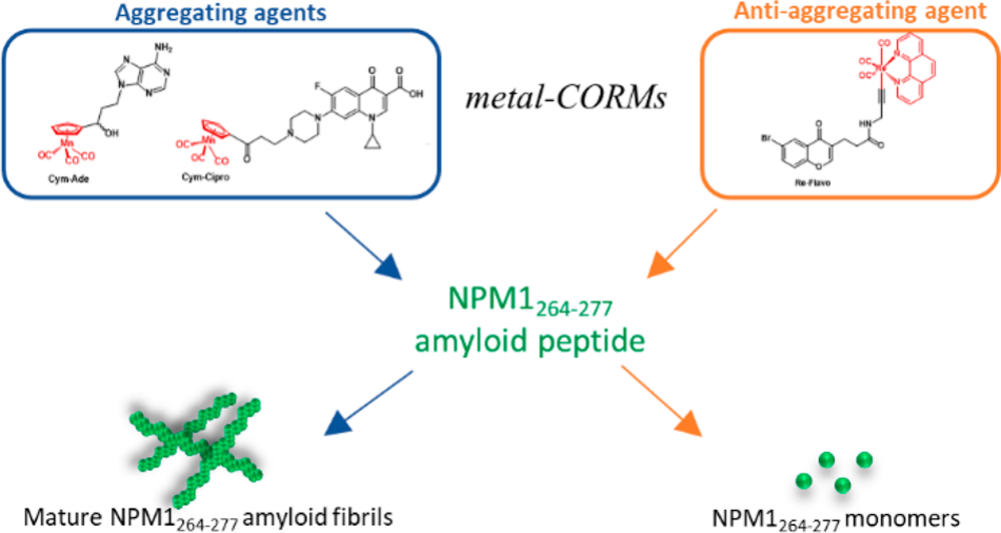

Neurodegenerative diseases are often associated with an uncontrolled amyloid aggregation. Hence, many studies are oriented to discover new compounds that are able to modulate self-recognition mechanisms of proteins involved in the development of these pathologies. Herein, three metal-complexes able to release carbon monoxide (CORMs) were analyzed for their ability to affect the self-aggregation of the amyloidogenic fragment of nucleophosmin 1, corresponding to the second helix of the three-helix bundle located in the C-terminal domain of the protein, i.e., NPM1_264–277_, peptide. These complexes were two cymantrenes coordinated to the nucleobase adenine (**Cym-Ade**) and to the antibiotic ciprofloxacin (**Cym-Cipro**) and a Re(I)-compound containing 1,10-phenanthroline and 3-CCCH_2_NHCOCH_2_CH_2_-6-bromo-chromone as ligands (**Re-Flavo**). Thioflavin T (ThT) assay, UV–vis absorption and fluorescence spectroscopies, scanning electron microscopy (SEM), and electrospray ionization mass spectrometry (ESI-MS) indicated that the three compounds have different effects on the peptide aggregation. **Cym-Ade** and **Cym-Cipro** act as aggregating agents. **Cym-Ade** induces the formation of NPM1_264–277_ fibers longer and stiffer than that formed by NPM1_264–277_ alone; irradiation of complexes speeds the formation of fibers that are more flexible and thicker than those found without irradiation. **Cym-Cipro** induces the formation of longer fibers, although slightly thinner in diameter. Conversely, **Re-Flavo** acts as an antiaggregating agent. Overall, these results indicate that metal-based CORMs with diverse structural features can have a different effect on the formation of amyloid fibers. A proper choice of ligands attached to metal can allow the development of metal-based drugs with potential application as antiamyloidogenic agents.

## Abstract 2

Three photoactivable metal-CORMs are able to modulate in a different way the self-aggregation mechanism of the peptide covering the second helix of the C-terminal domain of nucleophosmin 1: two of them act as aggregating agents and one of them acts as an antiaggregating agent, highlighting the key role of ligand systems in the development of metal-based drugs with potential application as antiamyloidogenic agents.

## Introduction

Metallodrugs have a wide clinically accepted range of applications in cancer^1^ and inflammatory diseases,^2^ but more recently, they are receiving particular attention as potential neurodrugs.^3^ The modulation of aggregation by metallodrugs is due to both direct and indirect interactions with amyloid polypeptides including coordination, oxidation, and hydrolysis of amino acid of monomers or small oligomers that hamper or increase the formation of large-size oligomers.^3^ Indeed, different metal complexes targeting amyloid systems showed promising potentialities as antiaggregation agents by exhibiting different mechanism of actions (MOAs) for their unique physicochemical properties: the chemical reactivity of complexes in terms of both kinetics and thermodynamics can be readily fine-tuned by central metal itself.^4^ Metal-based carbon monoxide-releasing molecules (CORMs)^5^ are an important class of metallodrugs that are able to transport and release CO and, hence, to provide therapeutic effects as anti-inflammatory^6^ and antiapoptotic agents.^7,8^ There is great interest for these compounds because of their ability to control the CO delivery to specific sites. CORMs can be activated by various external stimuli: change of solvent, presence of enzymes,^9^ thermal, pH or light variations,^10^ and oxidation.^11^ Among metal-CORMs,^12^ a terpyridine-containing Mn(I) complex was found to be able to release CO under the control of blue light irradiation,^13^ while two Ru(II) complexes, with bipyridines ligands functionalized with amides and an alkyne functionality or a green-fluorescent BODIPY (boron-dipyrromethene) dye, were found to be quickly internalized with accumulation at the endoplasmic reticulum and mitochondria.^13^ Interestingly, it has been demonstrated that CO can modulate the cleavage of human islet amyloid polypeptide (hIAPP) and beta-amyloid (Aβ) production by decreasing BACE1 expression, reducing Aβ levels and improving memory deficits in Alzheimer’s disease (AD) transgenic mice.^14^ Ru-based CORMs exhibit neuroprotective effects through a direct antiaggregating effect on Aβ_1–42_^15^ as well as several Pd(II)-square planar compounds with thiosemicarbazones (TSC) ligands repressed aggregation of Aβ_1–40_.^16^ The insertion of TSC ligands in a ferrocenyl complex coupled to a {Mn(CO)_3_} moiety produced a conjugated system able to release CO, when irradiated with ultraviolet light, with cytotoxic effects.^17^ Several studies report on “template metal complexes” as ferrocenyl, or cymantrenyl systems, bearing bioactive agents as additional ligands that demonstrated improved therapeutic effects.^18^ Indeed, three Ru(I) complexes containing triazolopyrimidines demonstrated more efficient cytotoxicity in cancer cells in comparison with traditional cisplatin, NAMI-A, and KP1019 compounds.^19^ Recently, cymantrenes received much attention^20^ since they are easily conjugated to small biomolecules as peptides^21,22^ and nucleobases (uracil, thymine, adenine).^23^

In early stages of the drug-discovery process in neurodegenerative diseases, often modulators of amyloid aggregation are screened employing protein/peptide amyloids which, even not strictly involved in neurodegeneration, are assumed as structural models of amyloids.^24−26^ In this context, nucleophosmin 1 (NPM1) is not a neurodegenerative protein but contains, in the second helix of its C-terminal domain, a well-characterized fragment, 264–277 peptide, able to act as an amyloid model.^27^

**Figure 1:**
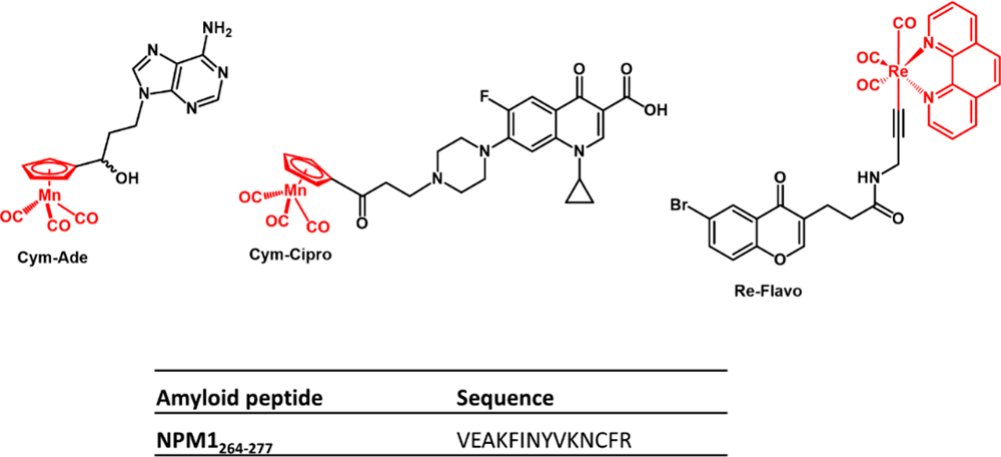
Chemical structures of the metal-based carbon monoxide-releasing molecules (CORMs) and sequence of amyloid model, investigated in this study.

In the recent past, we tested the ability of several Ru(II)-based CORMs to inhibit the amyloid aggregation NPM1_264–277_.^28^ Here, we investigated the ability to modulate NPM1_264–277_ aggregation of three metal-based CORMs: two cymantrenes conjugated to the nucleobase adenine (**Cym-Ade** in Figure 1) and to the antibiotic ciprofloxacin (**Cym-Cipro** in Figure 1), respectively, and a Re(I)-complex containing 1,10-phenanthroline and 3-CCCH_2_NHCOCH_2_CH_2_–6-bromo-chromone as ligands (**Re-Flavo** in Figure 1). Previous studies indicated that **Cym-Ade** adopts in the solid a bent conformation with the adenine and cyclopentadienyl planes perpendicular to each other and that it has antitrypanosomal activity against *T. brucei* strain (GI_50_ = 30.8 ± 3.5 μM).^29^**Cym-Cipro** is a *N*-alkyl cymantrenyl-ciprofloxacin conjugate that showed an antibacterial activity higher than that of ciprofloxacin alone since it acts following a dual MOA: (i) the inhibition of bacterial topoisomerase by ciprofloxacin and (ii) the generation of reactive oxygen species (ROS) caused by the organometallic moiety.^30^**Re-Flavo** displayed orange-red luminescence due to a ^3^MLCT excited state associated with the organometallic Re(CO)_3_(phen) fragment. Interestingly, it exhibited luminescent properties for bioimaging application: it can be readily internalized into HeLa cancer cells and accumulates in the cytoplasm.^31^

In the present study, we initially investigated the effects of CORMs on Thioflavin T (ThT) aggregation profile of NPM1_264–277,_ before and after ultraviolet irradiation of the analyzed samples. We have also assessed the direct formation of adducts between oxidized forms of amyloid peptide and CORMS by means of electrospray ionization mass spectrometry (ESI-MS) studies and deepened several aspects through time-dependent autofluorescence. For some of these CORMs, we evaluated the affinity for the amyloid peptide through UV titration and their preliminary effects on the amyloid cytotoxicity in SHSY5 cells. The effects on the morphologies of amyloid fibers were analyzed using scanning electron microscopy (SEM).

## Results and Discussion

### CORMs Stabilities and Their Effects on the Aggregation of NPM1_264–277_ through ThT Assay

**Figure 2:**
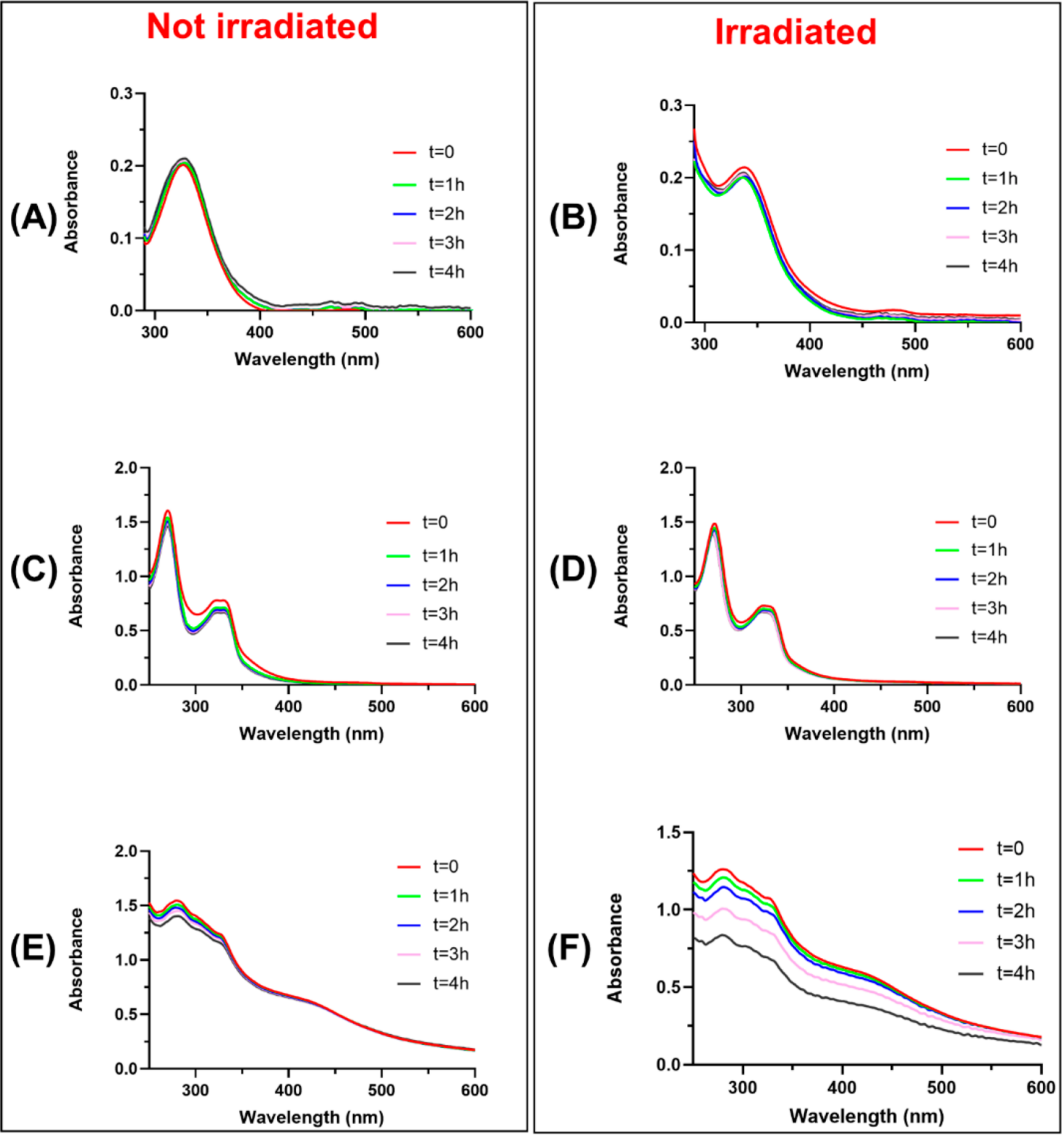
UV–vis spectra overtime of **Cym-Ade** (panels A and B), **Cym-Cipro** (panels C and D) and **Re-Flavo** (panels E and F), not irradiated (left) and irradiated at 365 nm (right) in 10 mM borate buffer at pH 8.5 (1% DMSO, v/v). Complex concentration was 200 μM for **Cym-Cipro** and 50 μM for **Cym-Ade** and **Re-Flavo**.

The stability of the CORMs reported in Figure 1 was analyzed by following UV–vis spectral profiles in 10 mM borate buffer at pH= 8.5,^27^ dimethyl sulfoxide (DMSO; 1% v/v), for 4 h. Samples were analyzed with or without irradiation at 365 nm, for 30 min^32^ (Figure 2). Spectra collected over time of not irradiated **Cym-Ade** appear superimposable (Figure 2A), while in the case of an irradiated sample, they are variable in the interval 3–4 h (Figure 2B). These features allow us to assign a common stability interval time of ∼2 h to irradiated and not irradiated **Cym-Ade**. Noticeably, by comparing spectra registered at t = 0 of irradiated and not irradiated **Cym-Ade**, a bathochromic shift of ∼20 nm is observed upon irradiation, since λ_max_ shifts from ∼327 to ∼348 nm (the overlay of spectra is reported in Figure S1A). This feature suggests a variation in the ligand field of the metal center. Spectra of not irradiated **Cym-Cipro** also do not change with time (Figure 2C), while those of the irradiated sample exhibit a slight decrease of absorbance after 1 h. This spectral variation can be due to a slight precipitation of the sample under the experimental condition. The irradiation of **Cym-Cipro** does not cause changes in the λ_max_ position, but only a slight decrease of intensity, as observable from the overlay of t = 0 spectra (Figure S1B). Similarly, not-irradiated **Re-Flavo** shows superimposable spectra over time (Figure 2E), while the irradiated complex (Figure 2F) exhibits a progressive decrease of absorbance (Figure 2F). As **Cym-Cipro**, the superimposition of t = 0 spectra shows only differences in the intensities (Figure S1C). For both **Cym-Cipro** and **Re-Flavo**, the interval time stability can be considered as ∼1.5–2 h.

**Figure 3:**
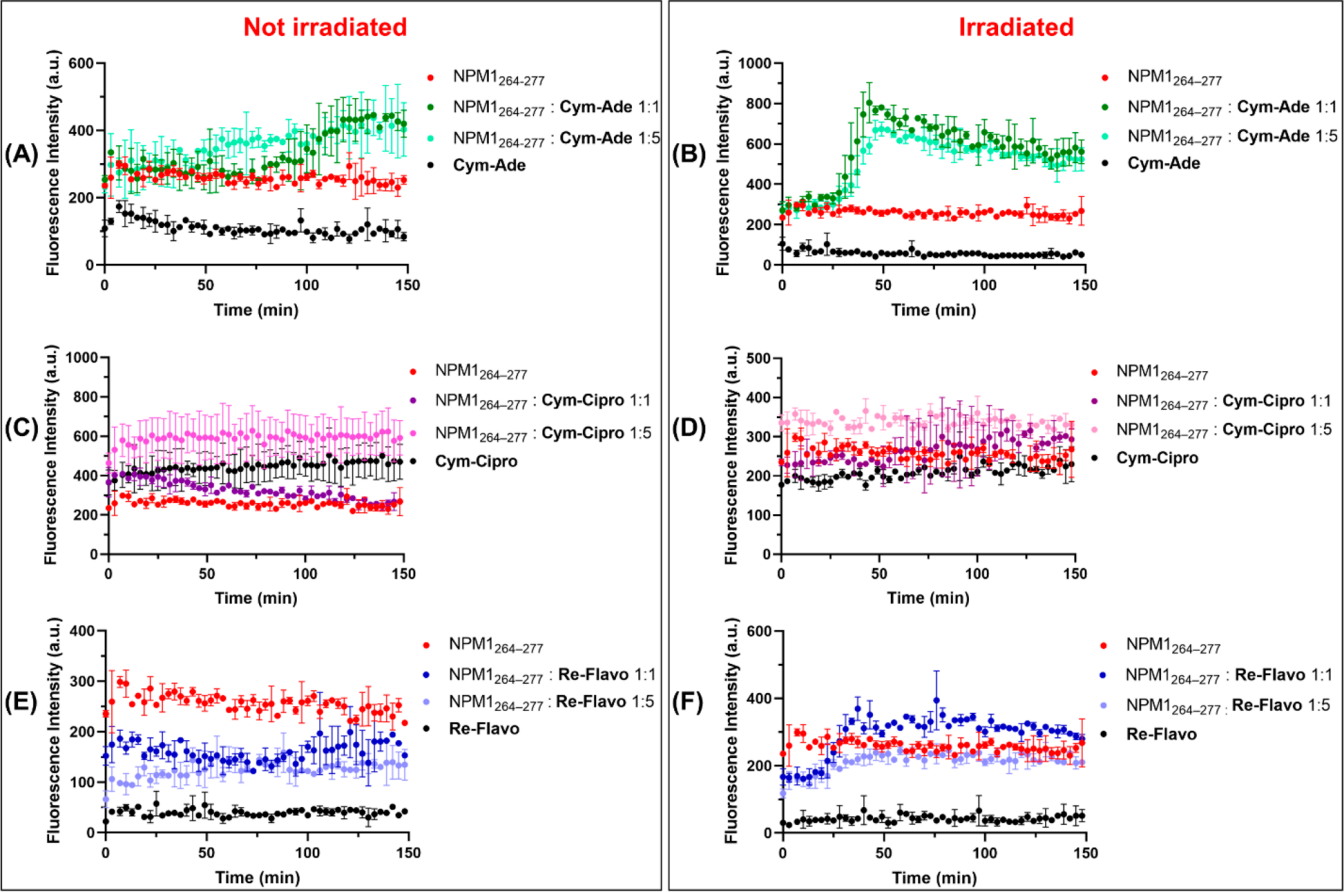
Overlay of time-courses of ThT fluorescence emission intensity of NPM1_264–277_ (100 μM) in the presence of **Cym-Ade** (panels A and B), **Cym-Cipro** (panels C and D), and **Re-Flavo** (panels E and F), at indicated peptide:metal complex molar ratios, without (left panel) and with irradiation at 365 nm (right panel) (10 mM borate buffer, 1% DMSO (v/v)). Results are representative of two independent experiments.

On the basis of stability results, we evaluated if metal complexes can affect the aggregation properties of NPM1_264–277_ using the ThT assay and monitoring its emission for 2 h. This assay allows us to analyze the kinetic of self-recognition process associated with amyloidogenesis. The overlays of time courses of the ThT fluorescence intensity, when NPM1_264–277_ was incubated with the three complexes, at different ratios, with and without irradiation, are reported in Figure 3.

The presence of the three metal complexes induces different effects on the aggregation profile of NPM1_264–277_, as also indicated by the comparison of t _1/2_ values that is the time at which fluorescence intensity reaches its half-maximum value.^33^

In particular, **Cym-Ade** (Figure 3A, B) causes an increase of ThT intensity and, thus, an enhancement of amyloid aggregation. This effect appears dose-dependent. Indeed, in the case of the not irradiated sample, the 1:1 NPM1_264–277_:**Cym-Ade** molar ratio implies a t_1/2_ = 110 min, while the 1:5 molar ratio a t_1/2_ = 55 min (Figure 3A). Conversely, after irradiation, the spectral profiles of the samples with different values of peptide:complex ratios are quite superimposable. It seems that the irradiation speeds the aggregation process, providing a t_1/2_ = 35 min (Figure 3B). In both conditions, the interaction of **Cym-Ade** with ThT is negligible.

**Re-Flavo** causes a reduction of the ThT intensity when it is not irradiated, acting as an antiaggregating agent, at both 1:1 and 1:5 NPM1_264–277_: **Re-Flavo** molar ratios (Figure 3E). After irradiation, **Re-Flavo** has an almost ignorable effect on the aggregation propensity of the peptide in the 1:5 NPM1_264–277_: **Re-Flavo** molar ratio, while it shows a slight enhancing-aggregating effect at the 1:1 NPM1_264–277_: **Re-Flavo** molar ratio, with a t_1/2_ = 25 min (Figure 3F). Control experiments indicate that ThT fluorescence emission intensity in the absence and in the presence of **Re-Flavo** are very similar.

Conversely, **Cym-Cipro** seems to interact with ThT, providing a nonzero signal that prevents the analysis of the effect of its presence on NPM1_264–277_ aggregation employing ThT assay (Figure 3C,D).

### ESI-MS Analysis of Adducts between NPM1_264–277_ and CORMs

**Figure 4:**
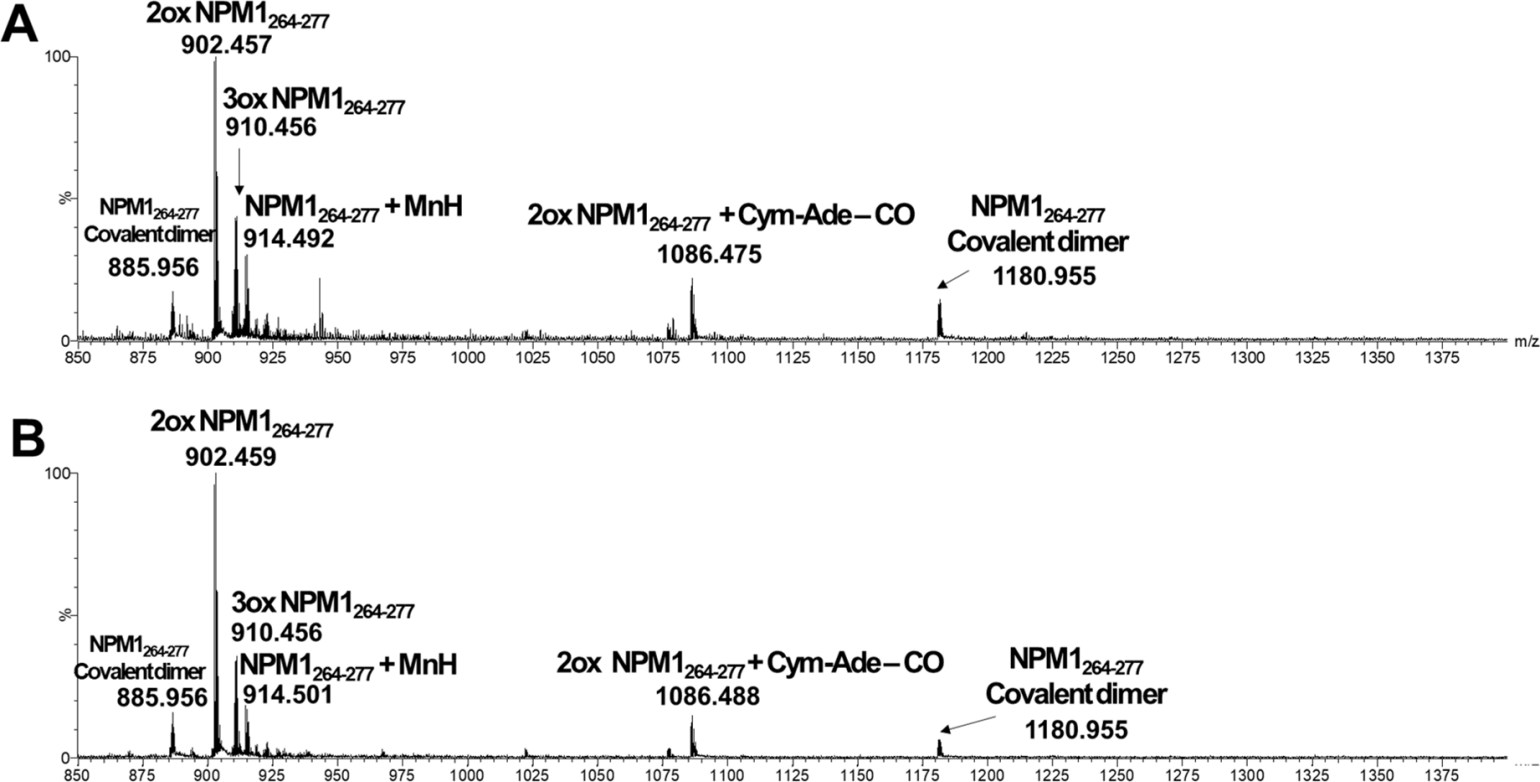
ESI-MS spectra of (A) not-irradiated, (B) irradiated **Cym-Ade** incubated with NPM1_264–277_.

**Figure 5:**
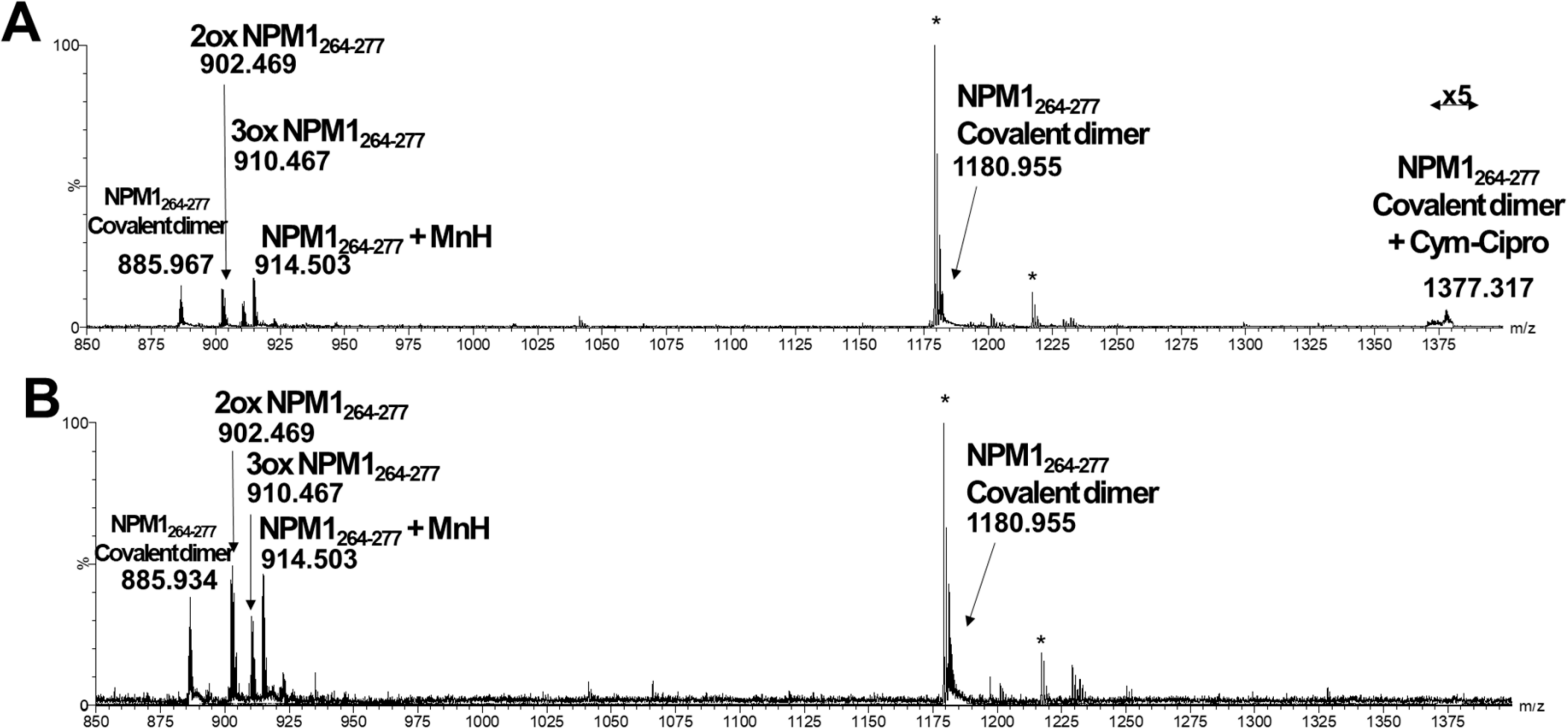
ESI-MS spectra of (A) not-irradiated, (B) irradiated **Cym-Cipro** incubated with NPM1_264–277_. The asterisk highlights the species present also in the MS spectrum of the **Cym-Cipro** alone.

**Figure 6:**
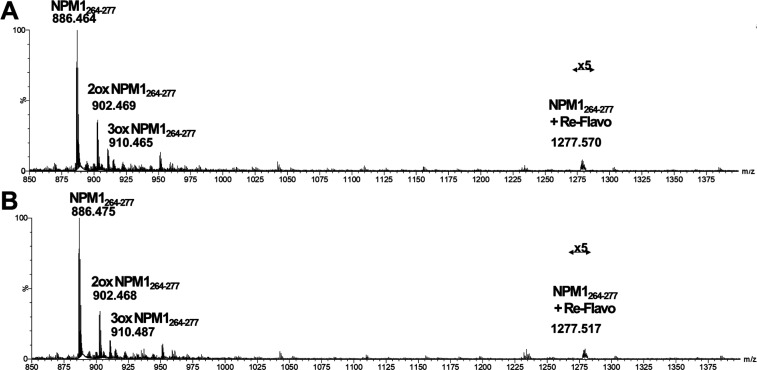
ESI-MS spectra of (A) not-irradiated, (B) irradiated **Re-Flavo** incubated with NPM1_264–277_.

We further analyzed the potential formation of adducts between CORMs and NPM1_264–277_ peptide through native ESI-MS analysis. In detail, complexes were added to the peptide, at the 1:5 molar ratio in both irradiated and not irradiated conditions, and the spectra are reported in Figure 4 for **Cym-Ade**, in Figure 5 for **Cym-Cipro** and in Figure 6 for **Re-Flavo**.

For Mn-complexes, no appreciable differences, between not irradiated (panel A) and irradiated (panel B) samples, are evident since a similar pattern of mass peaks is shown. First, the data are consistent with dual Cys^275^ oxidation, as demonstrated by the occurrence of intense signals at *m*/*z* 902.46 and 910.47 a.m.u., corresponding to dioxidated form of Cys as sulfinic acid,^34^ namely 2ox NPM1_264–277_, and to the trioxidized form of Cys as sulfonic acid, namely 3ox NPM1_264–277_^35^ (Table 1). The analysis of MSMS spectrum of the ion at *m*/*z* 902.46 a.m.u., of the sample containing irradiated **Cym-Ade** with NPM1_264–277_, confirmed that the oxidized residue was Cys (Figure S2). Furthermore, even if in traces, peaks for the mono-oxidized species are also present. The oxidative environment leads to the formation of the covalent dimer of NPM1_264–277_, containing a disulfide bridge, as demonstrated by the presence of the three- and quadruple-charged species (i.e., *m*/*z* 1180.955 and 885.956 a.m.u.). This is likely due to the ability of cymantrene compounds, in basic aqueous solution, to provide intermediate Mn oxidation states^29^ that in turn determine the aerobic oxidation of thiol group of Cys^275^.^36^ A common feature is represented by the formation of adducts of native NPM1_264–277_ with naked Mn ion, even in traces. Also in this case, the MS/MS analysis of the ion at *m*/*z* 914.50 a.m.u., confirmed that the thiol group of Cys^275^ is directly involved in binding to Mn (Figure S3). Despite these similarities, the two Cym-complexes behave differently in the adducts formation: for **Cym-Ade** a relevant species assigned to the 2ox NPM1_264–277_ bound to the metal complex lacking one CO group, at *m*/*z* 1086.49 a.m.u., was detected in both conditions (Figure 4), while for **Cym-Cipro**, the adduct corresponding to the NPM1_264–277_ dimer bound to the complex lacking the ciproxyl ligand is observable only when the sample is not-irradiated (Figure 5).

**Table 1:** Table of Main Observed Ions Relative to the Species Formed by the NPM1_264-277_ Peptide with Irradiated and Not-Irradiated **Cym-Ade**, **Cym-Cipro**, and **Re-Flavo**^a^

Compound	Experimental *m*/*z*	Charge	Theoretical *m*/*z*	Description
not-irradiated **Cym-Ade**	885.956	+4	885.948	NPM1_264–277_ covalent dimer
	902.457	+2	902.448	2ox NPM1_264–277_
	910.456	+2	910.448	3ox NPM1_264–277_
	914.492	+2	913.918	NPM1_264–277_ + MnH
	1086.475	+2	1086.068	2ox NPM1_264–277_ + Cym-Ade – CO
	1180.955	+3	1180.931	NPM1_264–277_ covalent dimer
irradiated **Cym-Ade**	885.956	+4	885.948	NPM1_264–277_ covalent dimer
	902.459	+2	902.448	2ox NPM1_264–277_
	910.456	+2	910.448	3ox NPM1_264–277_
	914.501	+2	913.918	NPM1_264–277_ + MnH
	1086.488	+2	1086.068	2ox NPM1_264–277_ + Cym-Ade – CO
	1180.955	+3	1180.931	NPM1_264–277_ covalent dimer
not-irradiated **Cym-Cipro**	885.967	+4	885.948	NPM1_264–277_ covalent dimer
	902.469	+2	902.448	2ox NPM1_264–277_
	910.445	+2	910.448	3ox NPM1_264–277_
	914.503	+2	913.918	NPM1_264–277_ + MnH
	1180.955	+3	1180.931	NPM1_264–277_ covalent dimer
	1377.317	+3	1377.265	NPM1_264–277_ covalent dimer + Cym-Cipro
irradiated **Cym-Cipro**	885.934	+4	885.948	NPM1_264–277_ covalent dimer
	902.469	+2	902.448	2ox NPM1_264–277_
	910.467	+2	910.448	3ox NPM1_264–277_
	914.503	+2	913.918	NPM1_264–277_ + MnH
	1180.955	+3	1180.931	NPM1_264–277_ covalent dimer
not-irradiated **Re-Flavo**	886.464	+2	886.448	NPM1_264–277_
	902.469	+2	902.448	2ox NPM1_264–277_
	910.465	+2	910.448	3ox NPM1_264–277_
	1277.570	+2	1277.448	NPM1_264–277_ + Re-Flavo
irradiated **Re-Flavo**	886.475	+2	886.448	NPM1_264–277_
	902.468	+2	902.448	2ox NPM1_264–277_
	910.487	+2	910.448	3ox NPM1_264–277_
	1277.517	+2	1277.448	NPM1_264–277_ + Re-Flavo

a Experimental and theoretical mass and charge were reported for each adduct.

Differently, in the presence of **Re-Flavo** in both samples, irradiated and not, the covalent dimer of NPM1_264–277_ is absent (Figure 6). Moreover, the levels of di- and three-oxidated species of the peptide are present but with lower intensity in comparison with the native peptide. The presence of the adduct of the peptide and the intact **Re-Flavo** complex (i.e., *m*/*z* 1277.953 a.m.u.) was detect even if in very small amount.

### Effects of Cym-Ade on the Aggregation of NPM1_264–277_: Spectroscopic Studies

**Figure 7:**
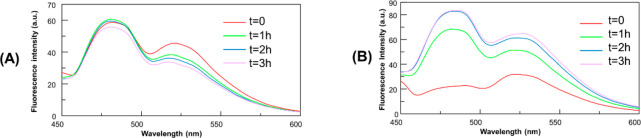
Overlay of fluorescence emission spectra, λ_exc_= 440 nm, of NPM1_264–277_ (100 μM) in (A) the absence and in (B) the presence of **Cym-Ade** at 1:5 NPM1_264–277_: **Cym-Ade** molar ratio (10 mM borate buffer at pH 8.5, 1% DMSO, v/v), under stirring.

To corroborate the enhancing-aggregation ability of **Cym-Ade**, amyloid generated autofluorescence of NPM1_264–277_^37,38^ was evaluated by monitoring emission spectra upon excitation at 440 nm over time (Figure 7). In detail, great differences between emission spectra of the amyloid peptide in the absence and in the presence of **Cym-Ade** are observed: indeed, while NPM1_264–277_ alone exhibits a decrease of fluorescence intensities at λ_max_ = 480 and 525 nm over time (Figure 7A), in the presence of **Cym-Ade**, a significant increase of intensity was observed (Figure 7B). Interestingly, the same experiment performed with **Cym-Cipro** shows only a slight increase in fluorescence intensity (Figure S4A). These findings suggest a great influence of **Cym-Ade** on the self-aggregation process of the amyloid peptide. Control experiments with **Cym-Cipro** and **Cym-Ade** alone reveal a negligible fluorescence of the two compounds upon excitation at 440 nm (Figure S4B and S5).

We have also evaluated the effects that the addition of NPM1_264–277_ has on the UV–vis spectrum of **Cym-Ade**. In both cases, an increase of absorbance at 320 nm was observed upon addition of the amyloid peptide (Figure S6). Saturated values of absorbance allow to estimate the half maximal effective concentration (EC_50_) values, that are equal to 4 ± 1 *10 μM for the not-irradiated sample and 9 ± 2 *10 μM for the irradiated one; these values are in line with similar systems.^39,28^

### Cellular Effects of Cymantrenyl CORMs on the Amyloid Cytotoxicity of NPM1_264–277_

**Figure 8:**
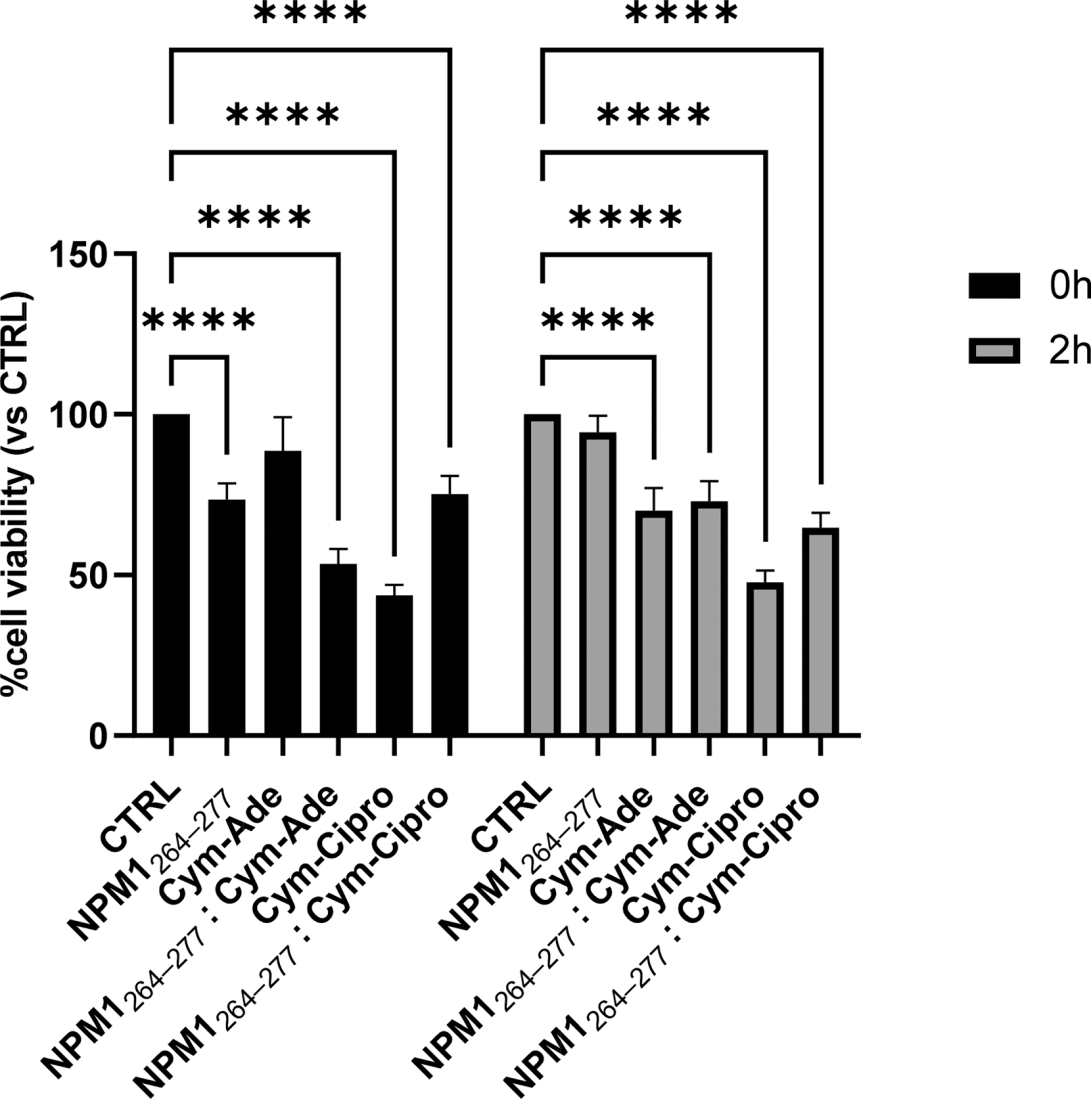
**Cytotoxic effect of molecules in SHSY5Y cells:** MTT assay of NPM1 _264–277_ alone and in the presence of **Cym-Ade** and **Cym-Cipro**, incubated under stirring at times t = 0, 2 h. We refer to the control untreated cells (CTRL) as 100% of viable cells. Statistical analysis was calculated by GraphPad Prism 9 by two-way Anova with Šídák’s multiple comparison test (***p = 0.0001).

We also evaluated preliminary effects of **Cym-Ade** and **Cym-Cipro** (at 1:5 peptide:metal compound molar ratio) on the cytotoxicity of NPM1_264–277_ on SHSY5 cells (Figure 8), through a MTT assay, performed incubating the cells with the peptides for 24 h. At t = 0 h, while the amyloid NPM1_264–277_ alone reduces cell viability of ∼25%, the adduct peptide with **Cym-Ade** exhibits a greater cytotoxic effect,^40^ leading to a reduction in cell viability of about 50%. This behavior is likely due to an increase of the amyloid character of the adduct **Cym-Ade**: NPM1_264–277_ that is in line with the ThT (see below) and SEM (see after) studies. After 2 h, this effect is strongly reduced. Contextually, **Cym-Ade** alone appears more cytotoxic than observed at t = 0 h. On the other hand, **Cym-Cipro** alone exhibits an intrinsic cytotoxic effect already at t = 0 that does not allow further speculations.

### Effects of CORMs on the Morphologies of NPM1_264–277_ Derived Fibers

On the basis of complexes stabilities, we evaluated potential effects of CORMs on the morphologies of NPM1_264–277_ fibers through scanning electron microscopy (SEM) experiments. All samples were obtained and analyzed after mixing at 1:5 NPM1_264–277_:complex molar ratio.

**Figure 9:**
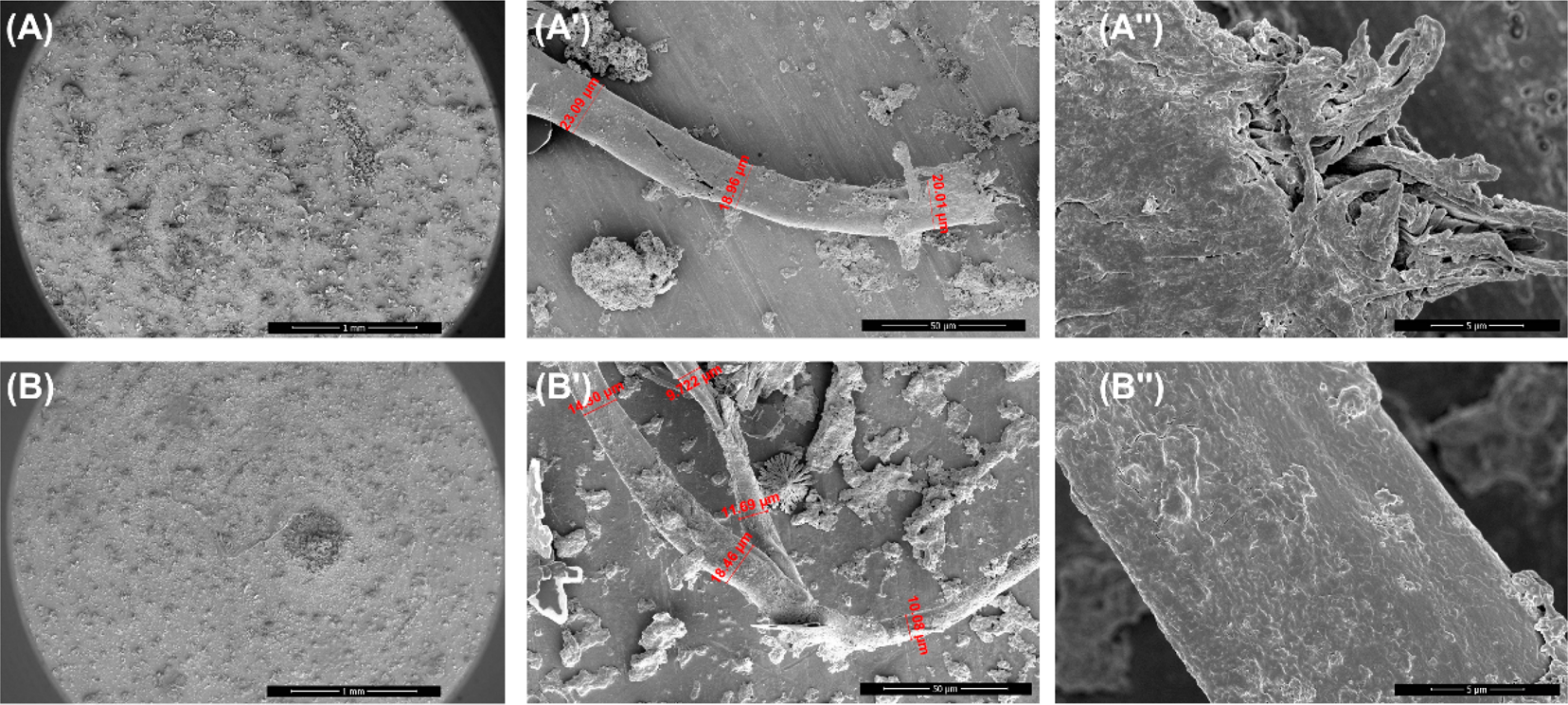
SEM micrographs of NPM1_264–277_ (100 μM) incubated with **Re-Flavo** (A-A′′) or **Cym-Cipro** (B–B′′) at 1:5 peptide:metal complex molar ratio. Overviews of the surface of samples at 1 mm (A,B), 50 μm (A′,B′) and 5 μm (A′′,B′′). In red, measured diameters: (A′) 23.09 μm, 18.96 μm, 20.01 μm; (B′) 14.30 μm, 18.46 μm, 10.08 μm, 9.722 μm, 11.69 μm.

**Cym-Cipro** did not exhibit any difference in irradiated and not irradiated conditions, and thus, only their not-irradiated samples were assayed and reported in Figure 9. The presence of **Re-Flavo** negatively affects the aggregative capacity of the amyloid peptide as already observed in ThT assay (Figure 3C) since only small and poorly formed fibers can be observed (Figure 9A). On the contrary, in the presence of **Cym-Cipro**, amyloid NPM1_264–277_ forms well-formed fibers that appeared longer, although slightly thinner in diameter, than those observed for NPM1_264–277_ alone (Figure 9B and Table 2).

**Table 2:** SEM Analyses. Average Diameter and Length of Fibers Obtained for NPM1 _264-277_ in the Presence and in the Absence of Metal Complexes

	Average Diameter (μm)	Average Length (μm)
NPM1 _264–277_^a^	22	1270
NPM1 _264–277_ + **Re-Flavo**	21	245
NPM1 _264–277_**+ Cym-Cipro**	15	1684
NPM1 _264–277_ + **Cym-Ade**	12	1294
NPM1 _264–277_ + **irradiated Cym-Ade**	18	987

a Ref (40).

**Figure 10:**
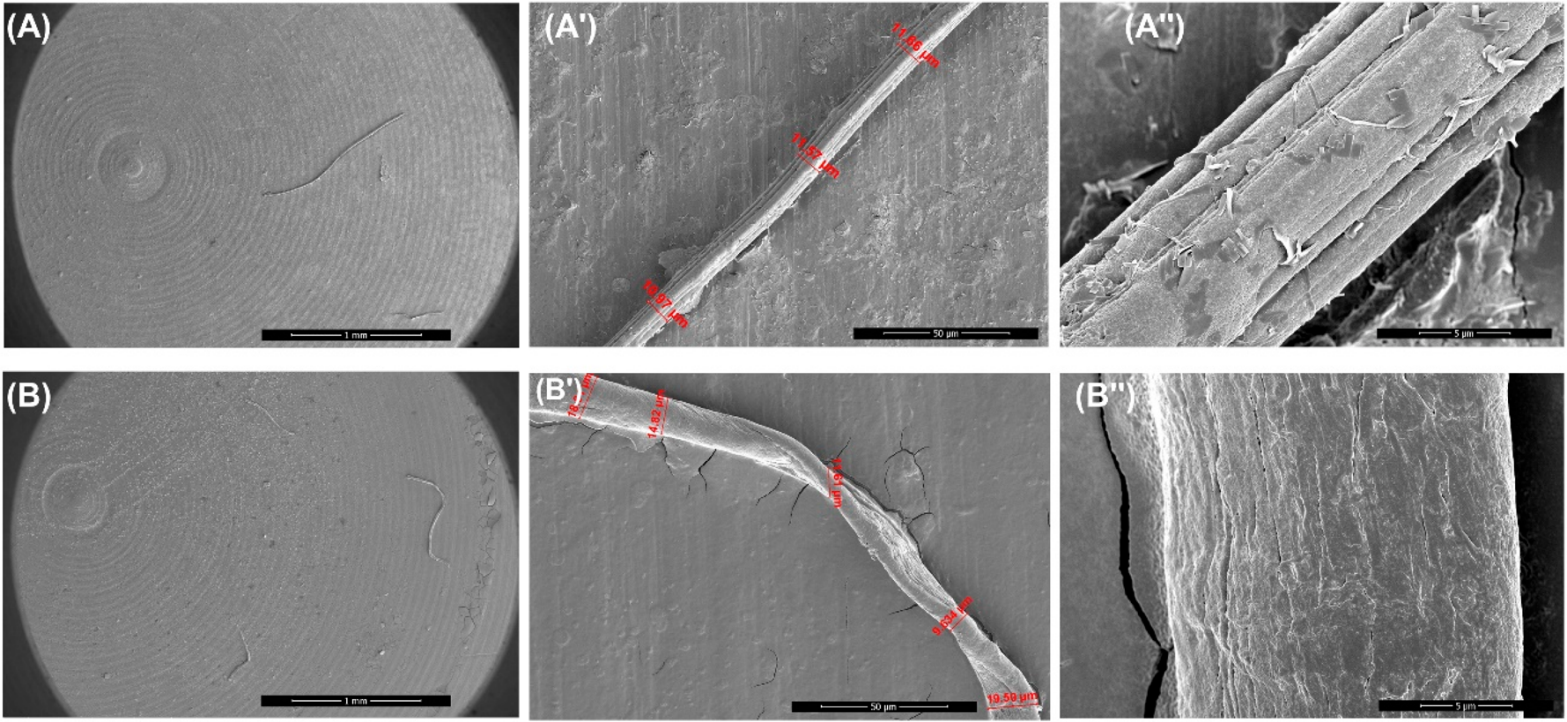
Morphology of assembled structures. SEM micrographs of NPM1_264–277_ peptide (100 μM) incubated with **Cym-Ade** not-irradiated (A-A′′) and irradiated (B–B′′) at 1:5 peptide:complex molar ratio. Overviews of the surface of samples at 1 mm (A,B), 50 μm (A′,B′) and 5 μm (A′′,B′′). In red, measured diameters: (A′) 11.66 μm, 11.57 μm, 10.97 μm; (B′) 18.01 μm, 14.82 μm, 11.61 μm, 9.634 μm, 19.50 μm.

The aggregates formed by NPM1_264–277_ in the presence of **Cym-Ade** were investigated using both irradiated and not-irradiated samples (Figure 10) to speculate about potential differences as evidenced by the ThT assay reported in Figure 3B. In fact, the aggregates resulting from two conditions appear quite different: fibers obtained using the not-irradiated sample are longer and stiffer than those formed by NPM1_264–277_ alone (Figure 10A-A′′), while those observed using the irradiated sample appear less ordered and thicker, as suggested by the presence of a twisted morphology (Figure 10B′), often observed in the case of other amyloid fibers.^41^

## Experimental Section

### Synthesis of the Metal Compounds and of the Peptide

**Cym-Ade**, **Cym-Cipro**, and **Re-Flavo** were prepared as previously reported^29−31^ as well as NPM1_264–277_, in the acetylated and amidated form.^27^ After purification, NPM1_264–277_ was treated with 1,1,1,3,3,3-hexafluoro-2-propanol (HFIP) and then stored at −20 °C until use.

### UV–vis Absorption Spectroscopy

The solution stability of the three CORMs was evaluated by collecting their UV–vis spectra in 10 mM borate buffer at pH 8.5 over 4 h. To collect the spectra, the compounds have been dissolved in DMSO (stock solutions 50 mM) and then added to the selected buffers to reach a final concentration of 200 μM. The DMSO final concentration was 0.4% (v/v). UV–vis spectra were registered on BioDrop Duo UV Visible Spectrophotometers (Cambridge, United Kingdom), at room temperature, using 1 cm path length cuvettes and the following parameters: 290–600 nm range, 200 nm/min, 2.0 nm bandwidth.

### Fluorescence Assays

ThT emission assay was carried out on fluorescence reader Envision 2105 (PerkinElmer) in black plates (96 well) under stirring. Measurements were collected every 2 min (λ_exc_ 440 nm and λ_emiss_ 485 nm) before (left) and after (right) 30 min of irradiation at 365 nm. Assays were performed at 25 °C employing a peptide concentration of 100 μM in 50 mM borate at pH 8.5, using a ThT final concentration of 50 μM, at different ratios with metal complexes (stock solutions 50 mM in DMSO, final DMSO concentration 1%). Autofluorescence experiments were carried out using 100 μM of NPM1_264–277_ alone or mixed with complexes at 1:5 peptide:metal compound molar ratio, at 25 °C in 50 mM borate at pH 8.5. λ_exc_: 440 nm λ_em_ range 450–600 nm.

For **Cym-Ade** samples t_1/2_ values were reported. t_1/2_ is the elapsed time at which F is equal to one-half of F _max_ following the fitting of data of F emission versus time through the empirical Hill equation^42^ as follows:
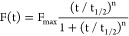
where F_max_ is the fluorescence intensity, and n is a cooperativity parameter.

### ESI-MS Analysis

The solution of NPM1_264–277_ peptide at a concentration of 10 μM in 15 mM AMAC (ammonium acetate) buffer pH = 7.0, was incubated with the complexes **Cym-Ade**, **Cym-Cipro**, and **Re-Flavo** in a molar ratio of 1:5, using both irradiated and not irradiated samples, and then analyzed by Q-TOF Premier (Waters, Milliford, MA, USA) mass spectrometer. The analyses were done by direct injection at 10 μL/min setting the source parameters at 3 kV for capillary voltage. The used acquisition range was from 100 to 2000 *m*/*z*, and the raw data were processed with MassLynx 4.1 software (Waters, Milliford, MA, USA). Mass calibration was performed on the same range by using 0.1% phosphoric acid as standard.

### Scanning Electron Microscopy

Samples (100 μL) containing NPM1_264–277_ (100 μM) alone or mixed with complexes at 1:5 peptide:metal compound molar ratio (10 mM borate buffer at pH 8.5, final DMSO concentration 1%), after 4 h of stirring, were dropped on stubs and introduced into chamber of field emission scanning (Nova NanoSem 450 FEI/ThermoFisher Scientific), to obtain Sem micrographs at 3.00 and 5.00 kV in high vacuum mode, with an Everhart Thornley Detector (ETD) and the Through the Lens Detector (TLD), as previously reported.^40^

### Cell Culture

Human SHSY5Y cell line was grown in a humidified atmosphere of 5% CO_2_ (37 °C) in Dulbecco’s Modified Eagle Medium (DMEM) (Microgem) supplemented with 10% heat inactivated fetal bovine serum (FBS) (GIBCO) and 2 mM l-glutamine, 50 ng/mL streptomycin, 50 units/mL penicillin.

### MTT Assay

Cells were seeded in triplicates in 96-well plates at a density of 7500 cells/well. NPM1_264–277_ (100 μM), **Cym-Ade** (500 μM), **Cym-Cipro** (500 μM), **Cym-Ade** + NPM1_264–277_ and **Cym-Cipro** + NPM1_264–277_ at 1:5 peptide:metal compound molar ratio were added (after 0 and 2h of stirring) to the cell cultures in triplicate and left for 24h at 37 °C in a humidified atmosphere of 5% CO_2_. These molecules were dissolved in DMSO that was used as control (1%). In the last 4 h of incubation, 3-(4,5-dimethylthiazol-2-yl)-2,5-diphenyltetrazolium bromide (MTT) was added to the cells.^43^ DMSO was then added to allow the reduction of MTT into formazan crystals by living cells. The absorbance was measured at 560 nm by Glomax Discover Microplate Reader (Promega Madison, WI, USA).

## Conclusions

Metallodrugs are unique and therapeutic agents that exert their bioactivity mostly through their coordination at the metal center and additional intermolecular interactions from the attached ligand(s).^44^ Recent examples are increasingly frequent in the literature, concerning metal complexes as antiaggregating agents of proteins involved in neurodegenerative diseases (NDDs).^45−49^ However, in drug-discovery related to NDDs, different approaches are generally employed; the most explored ones consist in the identification of inhibitors of amyloid formation to suppress toxic intermediates^28,50−56^ and in the search of agents able to accelerate the kinetics of conversion of toxic oligomers into fibers.^57^

In this work, we have analyzed the ability of three CORMs to modulate the aggregation of a model amyloid peptide system, the NPM1_264–277_ peptide. CORMs, in the interaction with biomolecules, can combine a coordination exchange mechanism, with the controlled release of the CO molecule. In detail, we investigated two Mn complexes with a cymantrenyl ligand frame with two different bioactive ligands, adenine in **Cym-Ade** and ciprofloxacin in **Cym-Cipro**, and one Rebased CORM, named **Re-Flavo**.

The formation of adducts between CORMs and NPM1_264–277_ and their effects on its aggregation propensity of the peptide were analyzed using various biophysical methods which provided different and unexpected results. ThT assay indicated that **Cym-Ade** enhances and speeds up NPM1_264–277_ aggregation properties especially if previously irradiated at 365 nm.

This effect was confirmed by SEM morphological analysis, which showed that in the presence of **Cym-Ade** NPM1_264–277_ forms amyloid fibers longer than those it forms in the absence of the metal compound, and by MTT assay, which revealed slightly higher cytotoxic effects of the amyloid in the presence of adenine-cymantrenyl complex. The structurally similar compound **Cym-Cipro** does not exhibit well-defined effects on ThT profiles, mainly because of a direct interaction with ThT dye, even if a slight lengthening effect on fibers was observed. However, ESI-MS analysis suggested that both cymantrenyl complexes interact with the peptide through a dual sequential MOA: as first the induction of oxidation of peptide sequence and, then, the coordination of the peptide to the Mn center through ligand exchange. The presence of oxo-species, containing sulfinic and sulfonic modifications of Cys, induce a more hydrophobic environment that favor self-recognition during amyloid aggregation. Furthermore, the peculiar geometry of **Cym-Ade**, with a reciprocal perpendicular disposition of adenine and cyclopentadiene planes, likely allows its accommodation in the growing β-sheet, facilitating the self-assembly and thus the aggregation. This mechanism can be kinetically favored by the availability of additional coordination positions around metal ion occurring in the irradiated sample. Conversely, **Re-Flavo** acts mainly as a slight inhibitor of aggregation as demonstrated by fluorescence and SEM studies and its antiaggregation effect might be explained with the lowest occurrence of oxidated species of the peptide, as demonstrated by the ESI-MS analysis. Overall, the presented data support the hypothesis that metal-CORMs with different ligand systems can be used to design in the future, metal-based drugs with potential application as antiamyloidogenic agents, since their direct translation as neurodrugs is hampered by the onset of intrinsic cytotoxicity already at 2 h of treatment.

## Supporting Information Available

Figure S1: Overlay of UV–vis spectra of **Cym-Ade**, **Cym-Cipro**, and **Re-Flavo**, before and after irradiation at 365 nm; Figure S2: MS/MS spectrum of 902.47 *m*/*z* signal in irradiated Cym-Ade complex (t = 0 h). In red is highlighted the diagnostic signal of Cys(2ox), Figure S3: MS/MS spectrum of 914.50 *m*/*z* signal in irradiated Cym-Ade complex (t = 0 h). In red are highlighted the diagnostic signals of Cys-MnH binding, Figure S4: Overlay of fluorescence emission spectra of Cym-Cipro in the presence of NPM1_264–277,_ at 1:5 NPM1_264–277_: Cym-Cipro molar ratio and alone; Figure S5: Overlay of fluorescence emission spectra of Cym-Ade; Figure S6: Normalized UV intensities at λ = 320 nm (not irradiated Cym-Ade) and λ = 340 nm (irradiated Cym-Ade) versus the Log of the concentration of NPM1_264–277_ (PDF)
