# Supplementary material for: Metal-Complexes Bearing Releasable CO Differently Modulate Amyloid Aggregation
Source: Inorg Chem. 2023 Jun 20;62(26):10470–80. doi: 10.1021/acs.inorgchem.3c01522 (PMC10324395; doi:10.1021/acs.inorgchem.3c01522)
Supplement: Supplementary file 1 — ic3c01522_si_001.pdf [file ic3c01522_si_001.pdf]

Supporting Information:

Metal-complexes bearing releasable CO differently modulate amyloid aggregation

*Sara La Manna[1], Valentina Roviello[2], Fabiana Napolitano[3], Anna Maria Malfitano[3], Vittoria Monaco[4,5], Antonello Merlino[4], Maria Monti[4,5], Konrad Kowalski[6], Łukasz Szczupak[6] and Daniela Marasco[1,]**

[1]Department of Pharmacy, University of Naples "Federico II", 80131, Naples, Italy

[2] Department of Chemical, Materials, and Industrial Production Engineering (DICMaPI), University of Naples Federico II, 80125 Naples, Italy.

[3]Department of Translational Medical Science, University of Naples "Federico II", 80131, Naples, Italy

[4]Department of Chemical Sciences, University of Naples "Federico II", 80126, Naples, Italy

[5]CEINGE Biotecnologie Avanzate S.c.a r.l., "University of Naples Federico II", 80131, Naples, Italy

[6] Faculty of Chemistry, Department of Organic Chemistry, University of Łódź, Tamka 12, 91-403 Łódź, Poland

Table of contents:

Figure S1: Overlay of UV-Vis spectra of **Cym-Ade**, **Cym-Cipro** and **Re-Flavo**, before and after irradiation at 365 nm.

Figure S2: MS/MS spectrum of 902.47 m/z signal in irradiated Cym-Ade +NMP1$_{264\text{-}277}$ sample (t=0h). In red is highlighted the diagnostic signal of Cys(2ox).

Figure S3: MS/MS spectrum of 914.50 m/z signal in irradiated Cym-Ade +NMP1$_{264\text{-}277}$ sample (t=0h). In red are highlighted the diagnostic signals of Cys-MnH binding

Figure S4: Overlay of fluorescence emission spectra of **Cym-Cipro** in the presence of NPM1$_{264–277,}$ at 1:5 NPM1$_{264–277}$: **Cym-Cipro** molar ratio and alone.

Figure S5: Overlay of fluorescence emission spectra of **Cym-Ade**, λ_{exct}= 440 nm.

Figure S6: Normalized UV intensities at λ=320 nm (not irradiated **Cym-Ade**) and λ=340 nm (irradiated **Cym-Ade**) versus the Log of the concentration of NPM1$_{264–277}$.

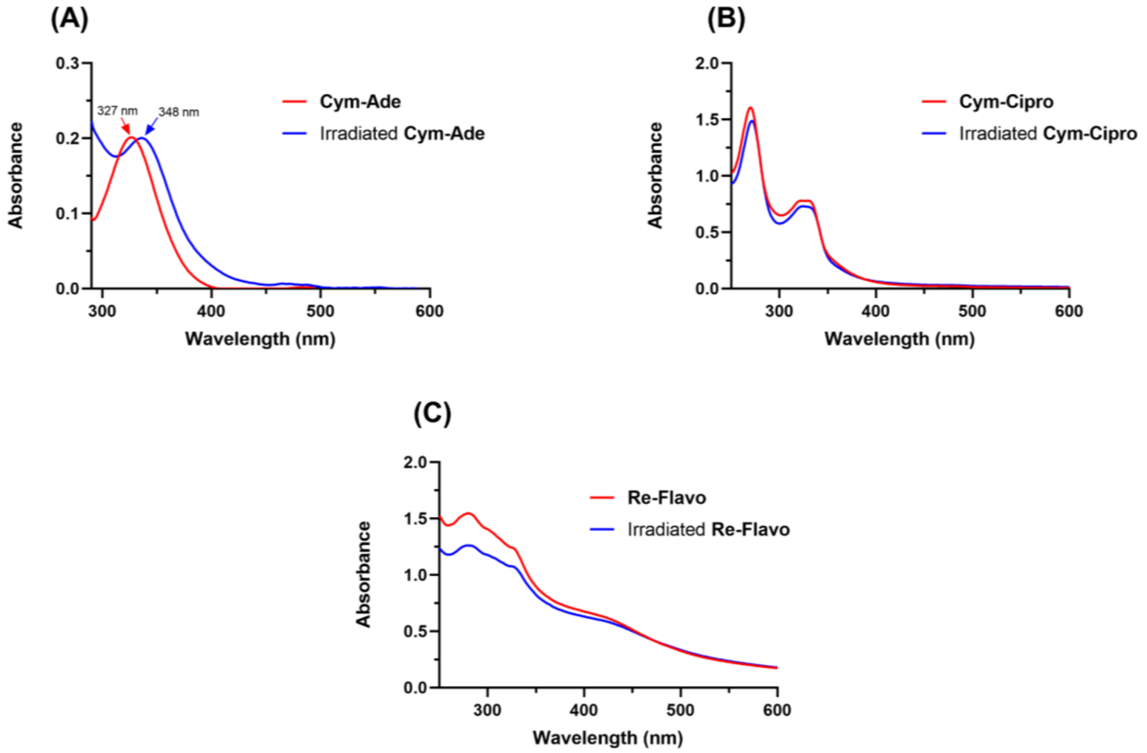

Figure S1. Overlay of UV-Vis spectra of A) **Cym-Ade**, B) **Cym-Cipro** and C) **Re-Flavo**, before (red line) and after (blue line) irradiation at 365 nm.

The MS/MS method was set up in a Data-Dependent Acquisition mode (DDA), with a full scan ranging from 375 to 1200 m/z range, followed by fragmentation of selected ions according to their intensities and charge states (+1, +2, +3, and multi-charges). Fragmentation spectra were manually analyzed to get the localization of modification (di-oxidation and MnH binding).

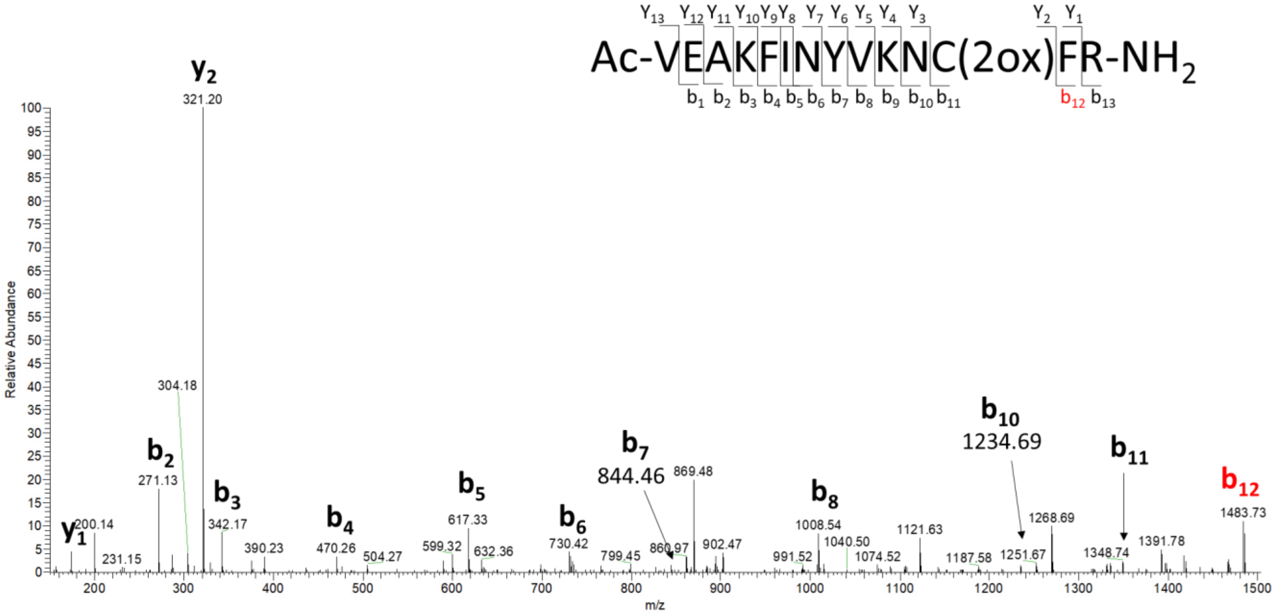

Figure S2: MS/MS spectrum of 902.47 m/z signal in irradiated Cym-Ade +NMP1$_{264-277}$ sample (t=0h). In red is highlighted the diagnostic signal of Cys(2ox).

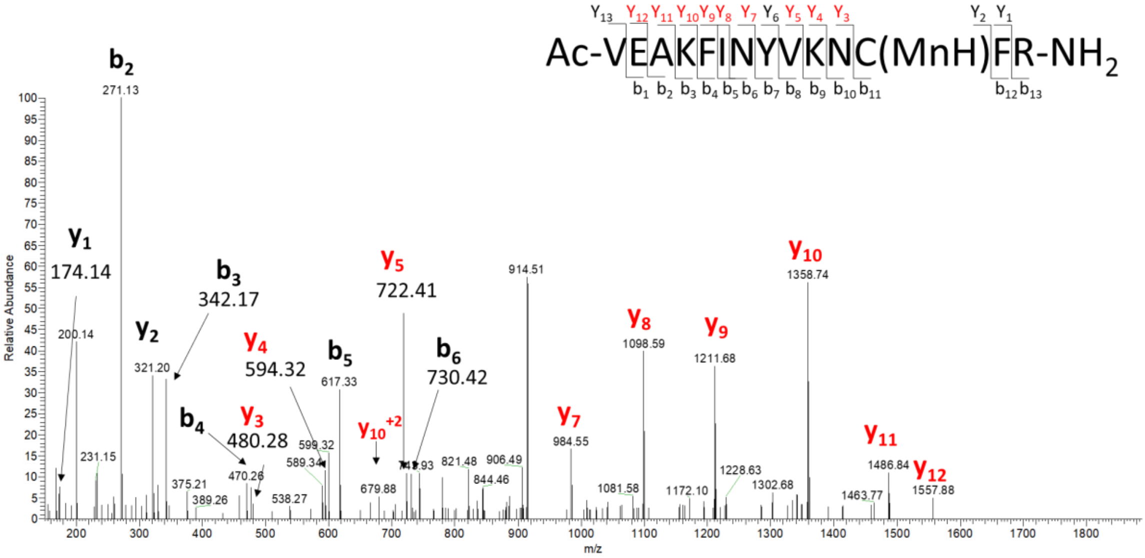

Figure S3: MS/MS spectrum of 914.50 m/z signal in irradiated Cym-Ade +NMP1$_{264\text{-}277}$ sample (t=0h). In red are highlighted the diagnostic signals of Cys-MnH binding

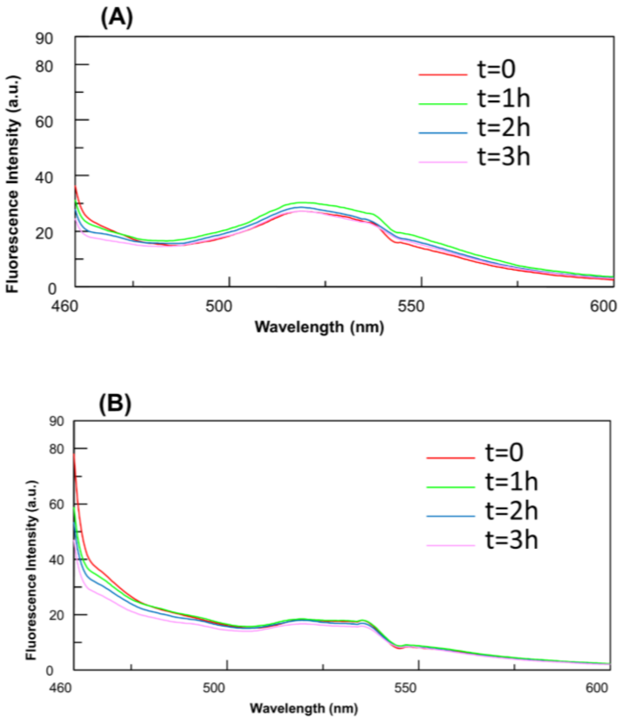

Figure S4. Overlay of fluorescence emission spectra, at indicated times, of **Cym-Cipro** (500 μM) A) in the presence of $NPM1_{264–277}$, at 1:5 $NPM1_{264–277}$: **Cym-Cipro** molar ratio and B) alone; λ_{exct}= 440 nm, under stirring.

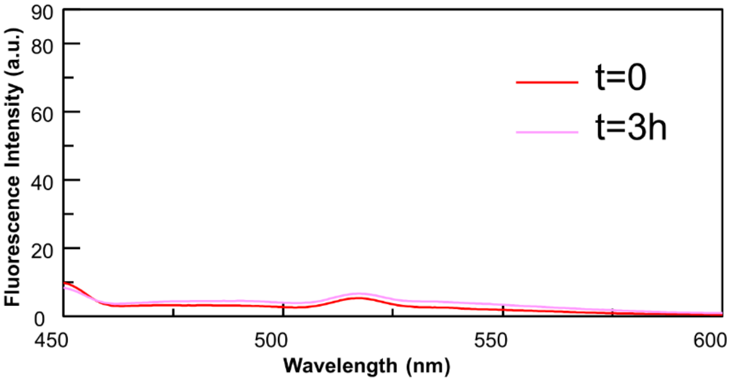

Figure S5. Overlay of fluorescence emission spectra of **Cym-Ade**, at indicated times, λ_{exct}= 440 nm, under stirring.

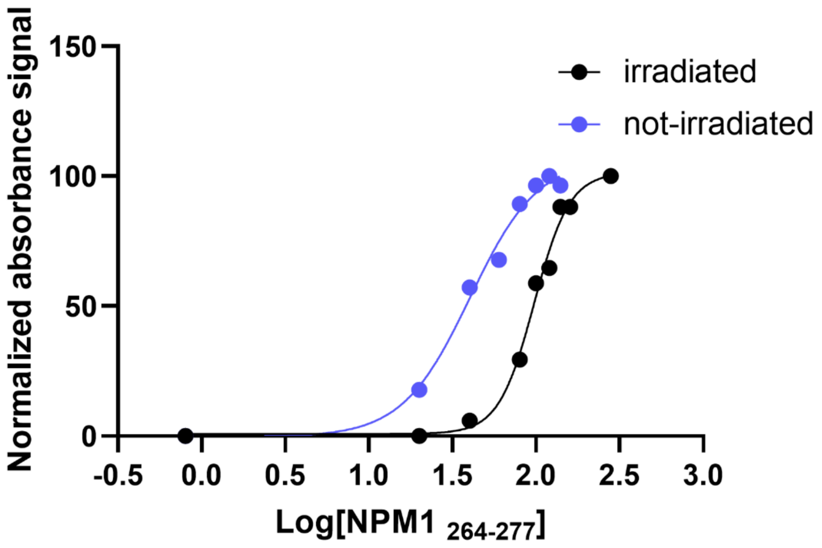

Figure S6. Normalized UV intensities at λ=320 nm (not irradiated **Cym-Ade**) and λ=340 nm (irradiated **Cym-Ade**) versus the Log of the concentration of NPM1$_{264–277}$.
